# Incidence and outcome of newly-diagnosed tuberculosis in schizophrenics: a 12-year, nationwide, retrospective longitudinal study

**DOI:** 10.1186/1471-2334-13-351

**Published:** 2013-07-29

**Authors:** Shu-Chen Kuo, Yung-Tai Chen, Szu-Yuan Li, Yi-Tzu Lee, Albert C Yang, Te-Li Chen, Chia-Jen Liu, Tzeng-Ji Chen, Ih-Jen Su, Chang-Phone Fung

**Affiliations:** 1 Institute of Clinical Medicine, National Yang-Ming University, School of Medicine, Taipei, Taiwan; 2 Division of Infectious Diseases, Taipei Veterans General Hospital, No. 201, Sec. 2, Shih-Pai Road, Taipei, 112, Taiwan; 3 National Institute of Infectious Diseases and Vaccinology, National Health Research Institutes, Miaoli County, Taiwan; 4 Division of Nephrology, Taipei Veterans General Hospital, Taipei, Taiwan; 5 Department of Medicine, Taipei City Hospital Heping Fuyou Branch, Taipei, Taiwan; 6 Department of Medicine, Chutung Veterans Hospital, Chutung, Taiwan; 7 Department of Psychiatry, Taipei Veterans General Hospital, Taipei, Taiwan; 8 Division of Psychiatry, School of Medicine, National Yang-Ming University, Taipei, Taiwan; 9 Institute of Public Health & School of Medicine, National Yang-Ming University, Taipei, Taiwan; 10 Division of Hematology and Oncology, Department of Medicine, Taipei Veterans General Hospital, Taipei, Taiwan; 11 Department of Internal Medicine, National Yang-Ming University Hospital, Yilan, Taiwan; 12 Department of Family Medicine, Taipei Veterans General Hospital, Taipei, Taiwan

**Keywords:** Schizophrenia, Tuberculosis, Incidence, Outcome

## Abstract

**Background:**

To control tuberculosis (TB), it is critical to identify at risk populations. Schizophrenia is recognized as an important risk factor for TB. However, previous studies have been confounded by comorbidities, and reports of TB infection outcomes are rare. Therefore, the current nation-wide study aimed to compare the adjusted incidence and outcome of TB diseases in schizophrenics and the general population.

**Method:**

Using the National Health Insurance Research Database from 1998 to 2009, this retrospective longitudinal study included 60,409 schizophrenics and general population matched for age, Charlson’s score, and comorbidities. Diagnosis of TB was based on the international classification of disease, ninth revision and use of anti-TB drugs. Unfavorable outcome for TB was defined as death, loss to follow-up, or use of anti-TB treatment for more than 9 months.

**Results:**

The adjusted incidence of TB in schizophrenics was significantly higher than in the general population [hazard ratio, 1.52; 95% confidence interval (CI), 1.29-1.79; p < 0.001; Kaplan-Meier log-rank test, p < 0.001]. Cox regression revealed age and male gender as risk factors for newly-diagnosed TB. The outcome of TB was comparable in schizophrenics and the general population [odds ratio (OR), 0.78; 95% CI, 0.55-1.09; p =0.144]. Logistic regression revealed a statistical trend for diabetes mellitus to predict poor outcome in schizophrenics with TB (OR, 2.30; 95% CI, 0.96-5.74; p = 0.062).

**Conclusions:**

Schizophrenics are at increased risk for TB, and screening may be warranted for those living in areas with high prevalence of TB.

## Background

Tuberculosis (TB) remains the most prevalent infectious disease and the major leading cause of death worldwide, accounting for more than 9 million new cases and 1.4 million deaths [1]. The World Health Organization’s “Global Plan to Stop TB” aims to eradicate this disease by 2050. An important strategy to control TB infection is to identify high-risk groups [2]. In addition to well-known risk factors of TB, such as diabetes mellitus (DM) and human immunodeficiency virus (HIV), many studies have demonstrated an increased incidence of TB in schizophrenics [3-8]. Schizophrenia is a debilitating mental disease affecting 1% of the general population [9], and it is often accompanied by physical illnesses [4,5,10] that may increase susceptibility to TB infection. However, previous studies have not adjusted for these confounding factors. In addition, outcomes for schizophrenics with concomitant TB have rarely been reported, though previous studies [11,12] reported an increased risk of death due to TB in patients with mental illness. To adjust for confounding factors and determine outcomes in schizophrenics requires studies with larger sample sizes and longer follow-up than those previously conducted. Therefore, we conducted a nation-wide, retrospective longitudinal study that spanned 12 years and included 120,818 people to compare the adjusted incidence and outcome of TB diseases in schizophrenics and the general population.

## Methods

### Data source

Taiwan regulations stipulate mandatory registry and treatment of TB via the Directly Observed Treatment Short Course (DOTS) program, which is monitored by the Center for Disease Control (CDC, Taiwan). DOTS workers and public health nurses follow TB cases to monitor compliance and side effects, and they report directly to the local health authority. National Health Insurance (NHI) provides coverage for anti-TB treatment and monitors the cost. After its implementation by the government under the principle of mandatory and universal enrollment in 1995, the NHI program covered 99% of the population of Taiwan. Computerized claims data for inpatient and outpatient care, including demographic data, treatment, and diagnosis by international classification of disease, ninth revision (ICD-9-CM) [13], were collected and stored in the NHI Research Database (NHIRD), which is run by the National Health Research Institute. We retrospectively retrieved inpatient and outpatient claims data from NHIRD for the period of January 1998 to December 2009 to compare the incidence and outcome of newly-diagnosed TB in schizophrenics and controls.

As the data set used in our study consisted of de-identified secondary data released to the public for research purposes, studies of this kind were exempt from full review by the institutional review board.

### Definitions

We performed a nation-wide study of schizophrenics and matched controls. The schizophrenic cohort comprised all patients who were admitted with schizophrenia (ICD-9-CM code 295.x) from January 1, 1998 to December 31, 2009. Schizophrenia diagnosis was further validated if it was coded by psychiatrists. The date of admission was considered the index date. Patients who were diagnosed with TB (ICD-9-CM codes 010.x to 018.x) before the index date were excluded.

Control subjects were selected from the Longitudinal Health Insurance Database dataset, which contains complete data of 1,000,000 randomly sampled beneficiaries from the original NHIRD. There were no significant differences in age or gender distribution in this sample of 1,000,000 beneficiaries and the original NHIRD. We excluded those patients who were diagnosed with schizophrenia and antecedent TB (ICD-9-CM codes 010.x to 018.x). However, there were significant differences in age, gender, and comorbid disease between schizophrenics and controls. Thus, we further refined our control cohort criteria by randomly selecting subjects to match schizophrenic patients on age (±2 years), gender, index date, Charlson’s score [13], chronic pulmonary disease, diabetes, and rheumatoid disease. To determine the incidence of TB, subjects were tracked from the index date until December 31, 2009, death, or diagnosis of TB. The diagnosis of new TB required the presence of ICD-9-CM code 010.x-018.x plus prescription of at least two anti-TB drugs (e.g., isoniazid, ethambutol, rifampin, pyrazinamide) for two months. Unfavorable outcomes included death, loss to follow-up, and prolonged treatment, which was defined as presence of ICD-9-CM code of TB for more than 9 months. Other comorbid diseases were retrieved by ICD-9-CM codes from the NHI database inpatient and outpatient datasets [13].

### Statistics analysis

Pearson chi-square tests were used for categorical variables, while independent t-tests and Mann–Whitney U tests were used for parametric and nonparametric continuous variables, respectively. The incidence of TB diseases was compared by Poisson distribution method, and the cumulative incidence of TB diseases was compared by the Kaplan-Meier method (log-rank test). Risk factors with p values of < 0.1 in univariate analysis were entered into the multivariate analysis. Multivariate Cox proportional hazard regression was performed using backward elimination to analyze independent risk factors for TB diseases. A p value of < 0.05 was considered significant.

## Results

### Baseline characteristics of schizophrenics

A total of 60,409 schizophrenics were included. The median follow-up period was 2,368 days (range, 1,449-3,295 days). Table 1 shows the clinical characteristics of this group. They were predominantly male (33,271, 55.1%), and the majority (93.4%) did not have any severe comorbidities (Charlson’s score < 3). The most prevalent underlying diseases were peptic ulcer diseases (11,549, 19.1%), chronic pulmonary diseases (10,096, 16.7%), and liver disease (7,796, 12.9%). A total of 60,409 controls were matched for gender, Charlson’s score, chronic pulmonary disease, DM, and rheumatoid disease (All p > 0.99).

**Table 1:** Baseline characteristics of schizophrenics and matched controls

**Characteristics**	**Schizophrenics (n = 60,409)**	**Controls (n = 60,409)**	**P value**
Age, median	35.4 (26.9-44.9)	35.3 (26.3-45.3)	0.001
Follow-up, days	2,368 (1,449-3,295)	2,296 (1,367-3,222)	
Male	33,271 (55.1%)	33,271 (55.1%)	>0.99
Charlson's score			
0	37,186 (61.6%)	37,186 (61.6%)	>0.99
1-2	19,216 (31.8%)	19,216 (31.8%)	>0.99
≥3	4,007 (6.6%)	4,007 (6.6%)	>0.99
Chronic pulmonary disease	10,096 (16.7%)	10,096 (16.7%)	>0.99
Diabetes	5,179 (8.6%)	5,179 (8.6%)	>0.99
Rheumatoid disease	934 (1.5%)	934 (1.5%)	>0.99
Peptic ulcer disease	11,548 (19.1%)	10,901 (18.0%)	<0.001
Liver disease	7,796 (12.9%)	8,584 (14.2%)	<0.001
Hypertension	7,071 (11.7%)	6,903 (11.4%)	0.131
Arrhythmia	6,420 (10.6%)	6,052 (10.0%)	<0.001
Dyslipidemia	4,988 (8.3%)	4,843 (8.0%)	0.127
Drug or substance abuse	2,725 (4.5%)	13 (0.02%)	<0.001
Chronic kidney disease	1,812 (3.0%)	2,048 (3.4%)	<0.001
Cancer	1,282 (2.1%)	1,934 (3.2%)	<0.001
Heart failure	819 (1.4%)	861 (1.4%)	0.303
Peripheral vascular disease	524 (0.9%)	484 (0.8%)	0.206
Myocardial infarction	229 (0.4%)	318 (0.5%)	<0.001
Hemiplegia or paraplegia	147 (0.2%)	205 (0.3%)	0.002
AIDS	53 (0.09%)	44 (0.07%)	0.361

Data are median value (interquartile range) for continuous variables and number of cases (%) for categorical variables. *AIDS:* acquired immunodeficiency syndrome.

### Higher incidence and risk factors of newly diagnosed TB cases in schizophrenics

**Figure 1:**
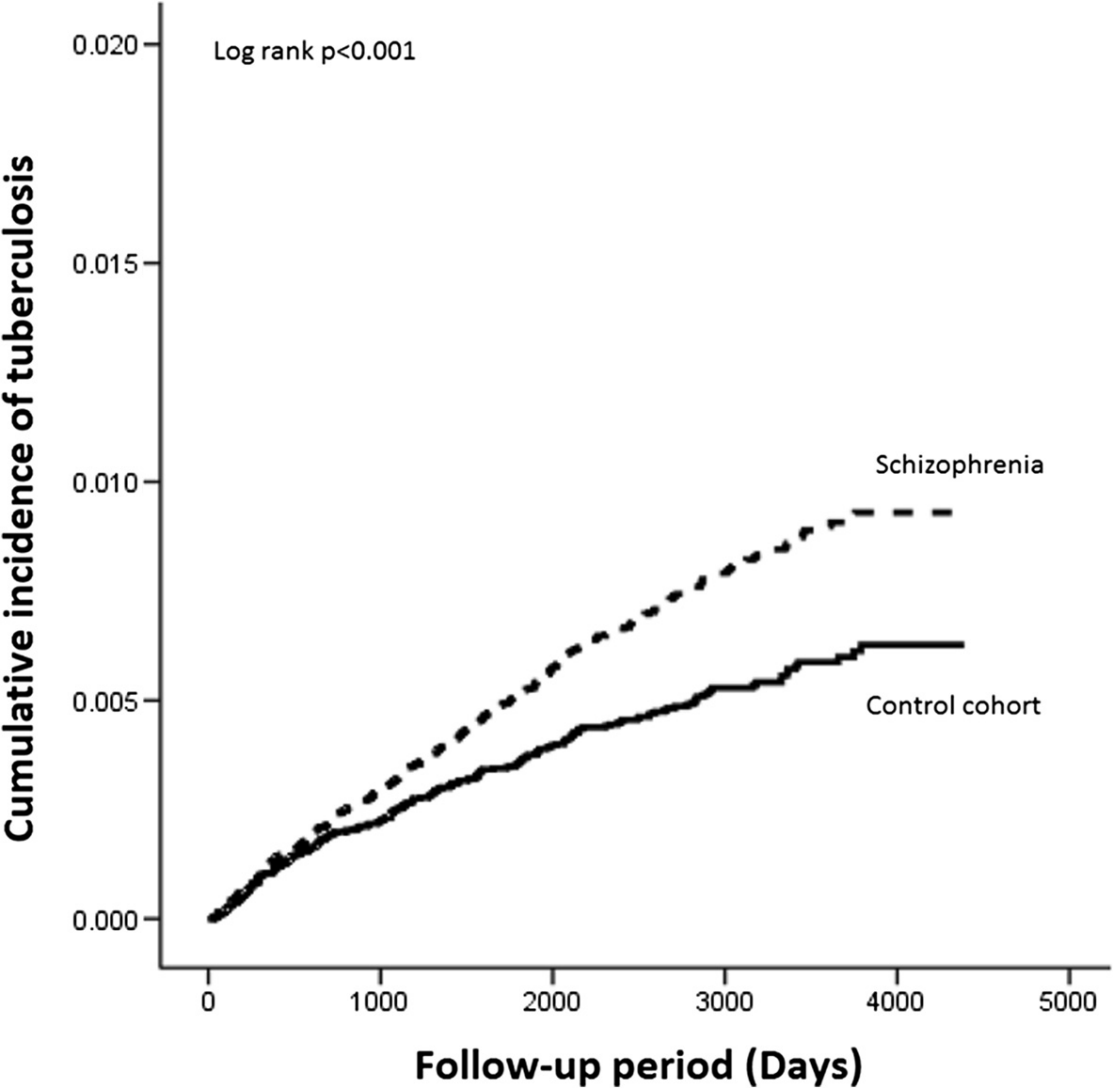
Kaplan-Meier plots of the cumulative incidence of tuberculosis between schizophrenics and control cohort.

There were 366 new TB cases among schizophrenics and 241 among controls during the total follow-up period of 771,657 person-years, as shown in Table 2. The crude hazard ratio (HR) of TB diseases in schizophrenics was 1.48 [95% confidence interval (CI), 1.26-1.74; p < 0.001]. After adjusting for all variables in Table 1, the HR of TB in schizophrenics was 1.52 (95% CI, 1.29-1.79; p < 0.001) compared with general population. As shown in Figure 1, Kaplan-Meier analysis also revealed a higher rate of newly-diagnosed TB in schizophrenics (log-rank test, p < 0.001). Most of the diseases were pulmonary in origin, occurring in 321 (87.7%) schizophrenics and 206 (85.5%) controls, as shown in Table 3 (p = 0.50). Compared with schizophrenics without TB (Table 4), those with TB tended to have advanced age, male gender, higher Charlson’s score, DM, myocardial infarction, and hypertension. Cox regression showed independent risks for newly-diagnosed TB to be age and male gender, while patients with hypertension were less likely to have new TB diseases.

**Table 2:** Incidence and crude and adjusted hazard ratio of newly-diagnosed tuberculosis in schizophrenics and controls

	**No.**	**Person-years**	**No. with TB**	**Incidence rate (per 10**^**5 **^**person-years)**	**Crude HR (95% CI)**	**Adjusted HR**^**1 **^**(95% CI)**
Total	120,818	771,657	607	78.7		
Schizophrenics	60,409	392,109	366	93.3	1.48* (1.26-1.74)	1.52* (1.29-1.79)
Controls	60,409	379,548	241	63.5	Reference	Reference

^1^Adjusted HR was adjusted for age, sex, gender, Charlson’s score and all comorbidities listed in Table 1. *TB:* tuberculosis, *HR:* hazard ratio, *CI:* confidence interval. *p < 0.001.

**Table 3:** Site of tuberculosis infection among schizophrenics and controls

**Site of infection**	**Schizophrenics (n = 366)**	**Controls (n = 241)**	**P value**
Pulmonary	321 (87.7)	206 (85.5)	0.502
Other respiratory	16 (4.4)	16 (6.6)	0.299
Skin, subcutaneous tissue, and lymph nodes	11 (3.0)	4 (1.7)	0.439
Primary tuberculosis	10 (2.7)	6 (2.5)	0.920
Miliary tuberculosis	3 (0.8)	2 (0.8)	>0.99
Meninges and central nervous system	2 (0.5)	1 (0.4)	>0.99
Intestines, peritoneum, and mesenteric glands	2 (0.5)	2 (0.8)	0.651
Bones and joints	1 (0.3)	2 (0.8)	0.566
Genitourinary system	0 (0)	2 (0.8)	0.157

Data are number of cases (%).

**Table 4:** Multivariate Cox regression of risk for newly-diagnosed TB in schizophrenics

**Characteristics**	**Univariate**			Multivariate*		
	**HR**	**95% CI**	**P value**	**HR**	**95% CI**	**P value**
Age, 1 year	1.05	1.04-1.06	<0.001	1.06	1.05-1.06	<0.001
Male	2.63	2.07-3.35	<0.001	3.13	2.46-3.99	<0.001
Charlson's score	1.18	1.07-1.29	0.001			
Diabetes	1.77	1.29-2.43	<0.001			
Chronic pulmonary disease	1.26	0.96-1.66	0.097			
Rheumatoid disease	1.51	0.71-3.18	0.283			
Myocardial infarction	4.70	1.94-11.37	0.001			
Heart failure	1.90	0.90-4.01	0.094			
Peripheral vascular disease	1.69	0.63-4.52	0.298			
Peptic ulcer disease	1.19	0.91-1.55	0.203			
Liver disease	1.05	0.76-1.45	0.773			
Hemiplegia or paraplegia	1.33	0.19-8.45	0.777			
Chronic kidney disease	1.14	0.61-2.13	0.693			
Cancer	1.62	0.87-3.05	0.131			
Hypertension	1.46	1.08-1.97	0.013	0.68	0.49-0.93	0.017
Dyslipidemia	1.16	0.79-1.71	0.45			
Arrhythmia	0.91	0.62-1.33	0.64			
Drug or substance abuse	1.44	0.90-2.32	0.13			
AIDS	0.05	0.00-2556713	0.74			

*HR:* hazard ratio, *CI:* confidence interval, *AIDS:* acquired immunodeficiency syndrome.

Only factors with P value less than 0.1 in the univariate analysis were entered into the multivariate analysis and only factors with P value less than 0.05 in multivariate analysis was presented.

### Comparable outcome of newly-diagnosed TB cases in schizophrenics and controls

Unfavorable outcomes occurred in 138 (37.7%) of 366 schizophrenics and in 105 (43.6%) of 241 controls with new TB diseases [crude odds ratio (OR), 0.78; 95% CI, 0.56-1.09; p =0.149]. During anti-TB therapy, 26 (7.1%) schizophrenics were lost to follow-up, 4 (1.1%) died, and 108 (29.5%) underwent treatment for more than 9 months. In the control group, 19 (7.9%) were lost to follow-up, one died (0.4%), and 85 (35.3%) underwent treatment for more than 9 months (All p > 0.05). After adjusting for comorbidities, schizophrenics and controls with new TB diseases had comparable outcomes (OR, 0.78; 95% CI, 0.55-1.09; p = 0.144). Multivariate analysis revealed a statistical trend for DM to predict poor outcome in schizophrenics (OR, 2.30; 95% CI, 0.96-5.74; p = 0.062, Additional file 1: Table S1).

## Discussion

Our nationwide study included a total of 60,409 schizophrenics and controls with a median follow-up duration of 6 years. After adjusting for underlying diseases, schizophrenics had a higher incidence of newly-diagnosed TB, but comparable outcomes to the general population. Independent risk factors for TB in schizophrenics were age and male gender. A statistical trend was found for DM as an independent risk factor for unfavorable prognosis in schizophrenics with TB.

The increased prevalence of TB in schizophrenics [3-8] may be related to different incidences of physical comorbidities [5], which could influence the likelihood of TB diseases. The relative risk of DM and colon cancer in schizophrenics has been reported as 1.5-2.0 [14] and 2.9 [15], respectively, compared with the general population, while infection rate of HIV in mentally ill patients was 4-23% compared with 0.3% for the general population in an area with high prevalence [16]. To our knowledge, this study has the largest number of schizophrenic subjects and is the first matched retrospective longitudinal study of its kind, which enabled adjusting for comorbidities. After adjustment, the incidence of newly-diagnosed TB was 1.5 times higher in schizophrenics compared to the general population, lower than previously reported [3-8]. The reason for the higher risk of TB in schizophrenics is unknown. Development of TB has been associated with several biological and social factors [1,17]. By adjusting biological factors, our study may indicate behavior and socioeconomic factors play an important role [17,18]. First, overcrowding and close contact is an established risk factor for TB [19]. Josoph et al. recently reported an outbreak within an assisted living facility for adults with mental illness [20], concluding that elevated risk for TB disease among this population was due to sustained transmission in crowded settings. Second, mentally ill patients have higher levels of substance abuse, such as smoking or alcohol use [21], which has been associated with active TB diseases [1]. Third, the low socioeconomic status of this vulnerable population [22,23] may predispose them to TB diseases [24]. Fourth, poor communication skills in this group may prevent or delay diagnosis and management of physical diseases [25]. Unfortunately, the anonymous and hospital-based nature of the NHI database precluded collection of data related to socioeconomic status and whether subjects lived in community-based facilities. Further studies are needed to evaluate the influence of these behavioral and socioeconomic factors on incidence of newly-diagnosed TB cases in schizophrenics.

The relative risk of death due to TB in patients with mental illness has been decreasing from 7.0 in the 1930s to 5.5 in the 1970s [11,12]. This has been attributed to improvements in treatment [3]. In our study, the rates of unfavorable outcomes for TB (mortality, loss to follow-up, and prolonged treatment) were comparable between schizophrenics and the general population after adjusting for comorbidities. This may be explained by coverage of 99% of the population by Taiwan NHI, as well as policies by the Taiwan CDC for enhancing diagnosis, follow-up, and treatment [26,27].

## Conclusions

In conclusion, schizophrenics had a higher incidence of newly-diagnosed TB than the general population. Fortunately, with advances in treatment and support from the national health care system, outcomes from TB were comparable in schizophrenics and the general population.

### Consent statement

As the data set used in our study consisted of de-identified secondary data released to the public for research purposes, the study was exempt from full review by the institutional review board.

## Abbreviations

CDC: Center for disease control; CI: Confidence interval; DM: Diabetes mellitus; DOTS: Directly observed treatment short course; HIV: Human immunodeficiency virus; HR: Hazard ratio; ICD-9-CM: International classification of disease, ninth revision; NHI: National health insurance; NHIRD: NHI Research database; OR: Odds ratio; TB: Tuberculosis.

## Competing interests

The authors declare that they have no competing interests.

## Authors’ contributions

SCK, SYL, YTL, and ACY managed the literature searches, designed the study, and completed the draft of the manuscript. YTC, CJL, and TJC collected the data, organized the database, undertook data analysis, and interpreted the data. TLC, IJS, and CPF monitored this program and contributed to manuscript revisions and approved the final version. All authors contributed to and have approved the final manuscript.

## Pre-publication history

The pre-publication history for this paper can be accessed here:

http://www.biomedcentral.com/1471-2334/13/351/prepub

## Supplementary Material

Additional file 1 Risk factor of unfavorable outcome of TB among schizophrenics. Click here for file

## References

[B1] Lawn SD Zumla AI Tuberculosis Lancet 2011 378 57 72 10.1016/S0140-6736(10)62173-3 21420161

[B2] WHO World Heath Organization. Global tuberculosis control: epidemiology, strategy, financing. 2009 Available at: http://www.who.int/tb/publications/global_report/2009/en/. Accessed August 2012

[B3] Ohta Y Nakane Y Mine M Nakama I Michitsuji S Araki K Tominaga Y Uchino J The epidemiological study of physical morbidity in schizophrenics–2. Association between schizophrenia and incidence of tuberculosis Jpn J Psychiatry Neurol 1988 42 41 47 3260975 10.1111/j.1440-1819.1988.tb01954.x

[B4] Baldwin JA Schizophrenia and physical disease Psychol Med 1979 9 611 618 10.1017/S0033291700033948 390590

[B5] De Hert M Correll CU Bobes J Cetkovich-Bakmas M Cohen D Asai I Detraux J Gautam S Moller H-J Ndetei DM Newcomer JW Uwakwe R Leucht S Physical illness in patients with severe mental disorders. I. Prevalence, impact of medications and disparities in health care World Psychiatry 2011 10 52 77 21379357 10.1002/j.2051-5545.2011.tb00014.x PMC3048500

[B6] Mishin VI Shevchuk EI Tsygankov BD Losev LV New-onset pulmonary tuberculosis patients with schizophrenia: course and efficiency of treatment Probl Tuberk Bolezn Legk 2008 6 6 10 18710046

[B7] Fisher II Bienskii AV Fedorova IV Experience in using serological tests in detecting tuberculosis in patients with severe mental pathology Probl Tuberk 1996 1 19 20 8907477

[B8] Zeenreich A Gochstein B Grinshpoon A Miron M Rosenman J Ben-Dov I Recurrent tuberculosis in a psychiatric hospital, recurrent outbreaks during 1987–1996 Harefuah 1998 134 168 172 9662903

[B9] Freedman R Schizophrenia. N Engl J Med 2003 349 1738 1749 10.1056/NEJMra035458 14585943

[B10] Dixon L Weiden P Delahanty J Goldberg R Postrado L Lucksted A Lehman A Prevalence and correlates of diabetes in national schizophrenia samples Schizophr Bull 2000 26 903 912 10.1093/oxfordjournals.schbul.a033504 11087022

[B11] Shinozaki H An epidemiologic study of deaths of psychiatric inpatients Compr Psychiatry 1976 17 425 436 10.1016/0010-440X(76)90045-6 1277817

[B12] Tsuang MT Woolson RF Fleming JA Causes of death in schizophrenia and manic-depression Br J Psychiatry 1980 136 239 242 10.1192/bjp.136.3.239 7388226

[B13] Deyo RA Cherkin DC Ciol MA Adapting a clinical comorbidity index for use with ICD-9-CM administrative databases J Clin Epidemiol 1992 45 613 619 10.1016/0895-4356(92)90133-8 1607900

[B14] De Hert M Dekker JM Wood D Kahl KG Holt RI Moller HJ Cardiovascular disease and diabetes in people with severe mental illness position statement from the European psychiatric association (EPA), supported by the European association for the study of diabetes (EASD) and the European society of cardiology (ESC) Eur Psychiatry 2009 24 412 424 10.1016/j.eurpsy.2009.01.005 19682863

[B15] Hippisley-Cox J Vinogradova Y Coupland C Parker C Risk of malignancy in patients with schizophrenia or bipolar disorder: nested case–control study Arch Gen Psychiatry 2007 64 1368 1376 10.1001/archpsyc.64.12.1368 18056544

[B16] Carey MP Carey KB Kalichman SC Risk for human immunodeficiency virus (HIV) infection among persons with severe mental illnesses Clin Psychol Rev 1997 17 271 291 10.1016/S0272-7358(97)00019-6 9160177

[B17] Kim S Crittenden KS Risk factors for tuberculosis among inmates: a retrospective analysis Public Health Nurs 2005 22 108 118 10.1111/j.0737-1209.2005.220204.x 15860066

[B18] Lienhardt C From exposure to disease: the role of environmental factors in susceptibility to and development of tuberculosis Epidemiol Rev 2001 23 288 301 10.1093/oxfordjournals.epirev.a000807 12192738

[B19] Vynnycky E Fine PE Interpreting the decline in tuberculosis: the role of secular trends in effective contact Int J Epidemiol 1999 28 327 334 10.1093/ije/28.2.327 10342699

[B20] Cavanaugh JS Powell K Renwick OJ Davis KL Hilliard A Benjamin C Mitruka K An outbreak of tuberculosis among adults with mental illness Am J Psychiatry 2012 169 569 575 10.1176/appi.ajp.2011.11081311 22684593

[B21] Kendrick T Cardiovascular and respiratory risk factors and symptoms among general practice patients with long-term mental illness Br J Psychiatry 1996 169 733 739 10.1192/bjp.169.6.733 8968631

[B22] Dohrenwend BP Levav I Shrout PE Schwartz S Naveh G Link BG Skodol AE Stueve A Socioeconomic status and psychiatric disorders: the causation-selection issue Science 1992 255 946 952 10.1126/science.1546291 1546291

[B23] Marwaha S Johnson S Schizophrenia and employment - a review Soc Psychiatry Psychiatr Epidemiol 2004 39 337 349 10.1007/s00127-004-0762-4 15133589

[B24] Olson NA Davidow AL Winston CA Chen MP Gazmararian JA Katz DJ A national study of socioeconomic status and tuberculosis rates by country of birth, United States, 1996–2005 BMC Public Health 2012 12 365 10.1186/1471-2458-12-365 22607324 PMC3506526

[B25] De Hert M Cohen D Bobes J Cetkovich-Bakmas M Leucht S Ndetei DM Newcomer JW Uwakwe R Asai I Moller HJ Gautam S Detraux J Correll CU Physical illness in patients with severe mental disorders. II. Barriers to care, monitoring and treatment guidelines, plus recommendations at the system and individual level World Psychiatry 2011 10 138 151 21633691 10.1002/j.2051-5545.2011.tb00036.x PMC3104888

[B26] Lo H-Y Yang S-L Chou P Chuang J-H Chiang C-Y Completeness and timeliness of tuberculosis notification in Taiwan BMC Public Health 2011 11 915 10.1186/1471-2458-11-915 22151346 PMC3260335

[B27] Li Y Tsai W Khan M Yang W Lee T Wu Y Kung P The effects of pay-for-performance on tuberculosis treatment in Taiwan Health Policy Plan 2010 25 334 341 10.1093/heapol/czq006 20207703

