# Supplementary material for: Incidence and outcome of newly-diagnosed tuberculosis in schizophrenics: a 12-year, nationwide, retrospective longitudinal study
Source: BMC Infect Dis. 2013 Jul 29;13:351. doi: 10.1186/1471-2334-13-351 (PMC3729604; doi:10.1186/1471-2334-13-351)
Supplement: Additional file 1 — Risk factor of unfavorable outcome of TB among schizophrenics. [file 1471-2334-13-351-S1.docx]

| Supplementary Table: Risk factor of unfavorable outcome of TB among schizophrenics | | | |
| --- | --- | --- | --- |
| Characteristics | OR | 95% CI | P value |
| Age, 1 year | 0.99 | 0.97-1.00 | 0.158 |
| Male | 0.90 | 0.53-1.54 | 0.711 |
| Charlson's score | 1.14 | 0.41-3.19 | 0.797 |
| Diabetes | 2.30 | 0.96-5.74 | 0.062 |
| Chronic pulmonary disease | 1.22 | 0.34-4.37 | 0.765 |
| Rheumatoid disease | 1.33 | 0.20-8.90 | 0.768 |
| Myocardial infarction | 0.74 | 0.06-8.73 | 0.814 |
| Heart failure | 0.44 | 0.05-4.05 | 0.466 |
| Peripheral vascular disease | 0.59 | 0.03-10.86 | 0.721 |
| Peptic ulcer disease | 0.90 | 0.26-3.06 | 0.866 |
| Liver disease | 0.82 | 0.22-3.04 | 0.769 |
| Chronic kidney disease | 1.51 | 0.23-9.95 | 0.668 |
| Cancer | 0.42 | 0.06-2.92 | 0.380 |
| Hypertension | 0.84 | 0.40-1.77 | 0.646 |
| Dyslipidemia | 0.55 | 0.19-1.58 | 0.266 |
| Arrhythmia | 0.45 | 0.17-1.21 | 0.112 |

 CI, confidence interval, OR, odds ratio.
